# Current understanding of epigenetics in atopic dermatitis

**DOI:** 10.1111/exd.14392

**Published:** 2021-06-01

**Authors:** Alina D. Schmidt, Cristina de Guzman Strong

**Affiliations:** ^1^ Division of Dermatology Department of Medicine Washington University in St. Louis School of Medicine St. Louis MO USA; ^2^ Center for Pharmacogenomics Department of Medicine Washington University in St. Louis School of Medicine St. Louis MO USA; ^3^ Center for the Study of Itch & Sensory Disorders Washington University in St. Louis School of Medicine St. Louis MO USA

**Keywords:** atopic dermatitis, inflammation, inflammatory skin diseases, T cells

## Abstract

Atopic dermatitis (AD) is an inflammatory skin disorder affecting up to 20% of the paediatric population worldwide. AD patients commonly exhibit dry skin and pruritus and are at a higher risk for developing asthma as well as allergic rhinitis. Filaggrin loss‐of‐function variants are the most widely replicated genetic risk factor among >40 genes associated with AD susceptibility. The prevalence of AD has tripled in the past 30 years in industrial countries around the world. This urgent public health issue has prompted the field to more thoroughly investigate the mechanisms that underlie AD pathogenesis amidst environmental exposures. Epigenetics is the study of heritable, yet reversible, modifications to the genome that affect gene expression. The past decade has seen an emergence of exciting studies identifying a role for epigenetic regulation associated with AD and at the interface of environmental factors. Such epigenetic studies have been empowered by sequencing technologies and human genome variation and epigenome maps. miRNAs that post‐transcriptionally modify gene expression and circRNAs have also been discovered to be associated with AD. Here, we review our current understanding of epigenetics associated with atopic dermatitis. We discuss studies identifying distinct DNA methylation changes in keratinocytes and T cells, eQTLs as DNA methylation switches that impact gene expression, and histone modification changes associated with AD‐related microbial dysbiosis. We further highlight the need for integrative and collaborative analyses to elucidate the impact of these epigenetic findings as potential drivers for AD pathogenesis and the translation of this new knowledge to develop newer targeted treatments.

## Graphical abstract

Atopic dermatitis, eczema, allergy, epigenetics, epigenome, DNA methylation, histone modification, phosphorylation, AcH3K9, miRNA, miR‐144, miR‐124, miR‐335, miR‐151a, ciRS‐7, S100A, KRT6A, KRT6B, OAS1, OAS2, OAS3, rs11740584, rs2299007, rs612529, CD4+ CLA+ T cells, NLRP2, hypermethylation, hypomethylation, whole genome bisulfite sequencing, HDAC, chromatin immunoprecipitation, eQTL, environment, microbiome, Staphylococcus aureus, Staphylococcus epidermidis, CpG, inflammation.

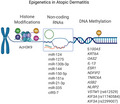

## 1 INTRODUCTION

Atopic dermatitis (AD) is a common and chronic inflammatory skin disorder characterized by skin barrier disruption and altered immune response.^1^ Patients with AD, also known as eczema, exhibit dry skin with an intense itch component (pruritus). Up to 20% of the paediatric population and 1%–3% of the adult population are affected worldwide.^2^, ^3^ Children who suffer from AD are at a greater risk of developing asthma and allergic rhinitis, collectively known as the “atopic march” of allergic hypersensitivity diseases.^4^ To date, at least 34 genetic loci and 46 genes have been associated with AD risk in diverse populations worldwide (see review^5^). Filaggrin (*FLG*) loss‐of‐function variants are the most widely replicated risk factor for AD and yet are specific to certain populations.^5^ Together, these studies reveal multiple genetic risk factors that contribute to AD pathogenesis. Additionally, the prevalence of AD continues to increase in industrial countries, suggesting increased exposure to environmental factors and associated lifestyles as potential risk factors for the disease.^6^, ^7^ These findings signify other non‐genetic contributors to AD pathogenesis that may include environmental interactions and epigenetic changes.^8^ Exciting studies in the past decade have begun to reveal epigenetic factors associated with atopic dermatitis pathogenesis, thus opening up new opportunities that target gene regulation as novel treatments for the disease.

Epigenetics impact the way DNA is transcribed from the genome.^9^ The etymology of epigenetics stems from epi‐ and genetics, with epigenetics derived from the Greek word for “on top of” or “around,” and conceived by the late developmental biologist, Conrad Waddington, to conceptually explain cell lineage specification.^10^ Thus, epigenetics constitutes cell‐specific alterations to the genome that are reversible and heritable and are not directly attributed to the DNA sequence. DNA methylation and the modification of histone proteins that bind to wrapped DNA are two well‐known epigenetic mechanisms of genome regulation (see review^11^). Here, we synthesize several decades of research leading up to our current conceptual understanding of histone modifications and DNA methylation as key drivers for epigenetic regulation.

We have come to understand that histone modifications in the genome are diverse. These epigenetic marks on the histones include acetylation, phosphorylation, methylation and ubiquitination that affect either the activation or the repression of transcription in a tissue‐specific manner. For example, common histone modifications such as H3K4me1 (mono‐methylation of the 4th lysine on histone 3) and H3K4me3 (tri‐methylation of the 4th lysine on histone 3) mark active enhancers and transcriptional start sites (TSS), respectively (see review^11^). Histone deacetylases (HDACs), histone acetyltransferases (HATs), histone methyltransferases (HMTs) and histone demethylases (HDMs) comprise a large class of enzymes that each catalyses its respective modification to the histone and hence “writes” an epigenetic mark to the DNA (see review^12^). Elucidation of these epigenetic histone marks can be determined using chromatin immunoprecipitation (ChIP) with antibodies specific for a given histone modification and subsequent quantitation using ELISA, qPCR and chip‐based assays. The coupling of ChIP with next‐generation sequencing (NGS), referred to as ChIP‐seq, provided a more comprehensive elucidation of these epigenetic modifications in numerous cell types and tissues—enabling genome‐wide discoveries in a more high‐throughput manner.^13^

DNA methylation more commonly represses transcription by generating a “silencing” mark to the genome (see review^14^). This methylation process is catalysed by DNA methyltransferases (DNMTs) that transfer a methyl group at the fifth carbon of cytosine resulting in 5‐methylcytosine (5mC) (see review^15^). 5‐mC methylation is typically found at CpG sites, comprised of repeats of cytosine followed by guanine residues, and often near transcriptional start sites (TSS), enhancers and insulators. Several methods exist to capture and detect DNA methylation. These methods include genomic DNA preparations using chemical bisulphite conversion, methylation‐sensitive enzymatic cleavage or immunoprecipitation of chromatin bound by methylcytosine‐specific antibody (ChIP). These regions are then quantitated using PCR‐based, chip‐based analyses or downstream NGS.^16^, ^17^, ^18^ Bisulphite conversion is considered the gold standard to DNA methylation; it deaminates unmethylated cytosine to uracil and discriminates between 5‐mC and 5‐hydroxymethylcytosine (5‐hmC) and can be coupled with high coverage whole‐genome bisulphite sequencing (WGBS).^19^, ^20^, ^21^ The range and sensitivity of DNA methylation detection methods provide multiple opportunities for both hypothesis‐testing and discovery‐based studies in AD.

The development of these key epigenetic concepts and technologies and the application to numerous cell and tissue types were pivotal for many laboratories and the Encyclopedia of DNA Elements (ENCODE) Project to successfully elucidate regulatory elements on a genome‐wide scale.^22^ In doing so, it provided the necessary tools and genomic maps that promulgated epigenetics to the forefront of mechanistic investigations in biology and disease. Indeed, epigenetics has been firmly established to greatly impact cancer development and cell lineage specification (see reviews^23^, ^24^, ^25^). This has since motivated parallel studies to investigate the role of epigenetics on the pathogenesis of inflammation.^26^, ^27^ Here, we discuss our current state of understanding of epigenetics in the common inflammatory skin disease, atopic dermatitis.

## 2 DNA METHYLATION IN ATOPIC DERMATITIS

A number of pioneering studies sought to investigate DNA methylation changes in AD and determine a mechanistic impact on pathogenic gene expression. Rodríguez et al were the first to investigate differences in methylation patterns in AD cases in contrast to healthy controls.^28^ The authors performed chip‐based studies and analyses to identify changes in gene expression and DNA methylation at CpG sites in AD lesional, AD non‐lesional and healthy control epidermis, and in T and B cells and whole blood. CpG methylation appeared to be unaffected in T and B cells and whole blood. However, CpG methylation changes and correlating gene expression were identified in the lesional epidermis of AD patients compared with that of healthy controls. Increased expressions for *S100A2*, *S100A7*, *S100A8*, *S100A9* and *S100A15* were observed, yet a methylation change was only found for *S100A5* that was hypermethylated. Moreover, *KRT6A* and *KRT6B* expressions were increased in keratinocytes but decreased methylation was only observed for *KRT6A*. Additionally, increased expressions for *OAS1*, *OAS2* and *OAS3* in innate immune cells were identified but only *OAS2* exhibited decreased methylation. Together, this study highlighted key DNA methylation changes that were specific to keratinocytes and innate immune cells in AD pathology.

Methylation studies in immune cell types have also revealed important findings for epigenetic patterns observed in AD. An early study performed genome‐wide CpG methylation profiling (methyl‐CPG‐binding domain (MBD)‐2b bound chromatin and downstream NGS) in CD4+ naive T cells in both AD and psoriasis patients.^29^ Increased methylation was found in 121 gene promoter regions in psoriasis patients versus controls, but no pair‐wise methylation differences were discovered in AD patients. However, a more recent study that investigated additional CD4+ T cell subsets and CD8+ T cells identified DNA methylation changes that were cell type‐specific.^30^ DNA methylation differences in CD4+, CD4+CD45RA+ naïve, CD4+CLA+ and CD8+ T cells were determined using a chip‐based assay in an AD case‐control cohort. Ten hypomethylated and 25 hypermethylated genes were specifically found in AD patients in CD4+ CLA+ T cells and not in the other cell types. Many of the genes are involved in immune inflammation and cytokine signalling pathways (*ARHGEF3*, *ASB2*, *DAPP1*, *IL10RA*, *PDE4A*, *SH2B3*, *STIM1* and *TOX2*). A direct relationship for decreased upstream methylation of *IL13* and increased *IL13* expression was identified, suggesting potential epigenetic regulation for *IL*‐*13*, a known Th2 marker for AD.^31^ miRNA profiling in CD4+CLA+ T cells and subsequent integrative analysis with DNA methylation levels next identified 16 differential expressed miRNAs, 4 of which were validated by qPCR (miR‐21‐3p, miR‐130b‐3p, miR‐150‐5p and miR‐1275). Pathway analysis further revealed 4 upregulated and differentially methylated genes (*ESR1*, *NDFIP2*, *ASB2* and *TNRC6A)* targeted by the upregulated miRNAs in AD patients. Together, these studies highlight both distinct methylation changes and correlative gene expressions in the pathogenically relevant CD4+ T cell subset, as well as for Th2 AD maker IL‐13, and the intricacies of multiple epigenetic regulators in AD. We further discuss the role of miRNA regulators later in this review.

Another important study examined the underlying DNA methylation differences in whole blood for nucleotide‐binding oligomerization and pyrin domain‐containing receptor (*NLRP*) genes that were differentially expressed in early‐onset AD compared with controls.^32^ *NLRP*s are known to modulate the innate immune response by activating NF‐kB activity.^33^ In this study, the authors identified decreased *NLRP2* expression in 1‐year‐old children with AD in contrast to age‐matched healthy controls. Whole‐genome bisulphite sequencing of cord blood samples obtained from a subset of these patients at birth was subsequently performed to determine DNA methylation changes. *NLRP2* promoter hypermethylation was identified and correlated with the decreased *NLRP2* expression levels observed at both time points (birth and one year of age) in AD children compared with healthy controls.^32^ This correlation was also associated with increased blood *IL*‐*10* in AD individuals. Mediation analysis for these same early‐onset AD children identified prenatal benzene exposure (a proxy for tobacco smoke) as a potential environmental trigger for the observed *NLRP2* promoter hypermethylation and decreased expression of *NLRP2*. This study provided new insight linking the impact of environmental exposure and associated methylation changes for predicting AD risk and longevity.

Several key papers investigating DNA methylation at distinct CpG sites have also revealed mechanistic insights for several genes involved with AD pathogenesis. In one study, rs612529‐T in the *VSTM1 (SIRL*‐*1)* promoter was reported to be significantly associated with allele‐specific, increased *SIRL*‐*1* expression in monocytes in Chinese and Caucasian healthy cohorts.^34^ The SNP is located within a CpG site with preferential YY1 and PU.1 transcription factor binding to the T allele. PU.1 is known to recruit Tet2 demethylase,^35^ and this finding led the authors to hypothesize allele‐specific demethylation of the *VSTM1* promoter resulting in increased *SIRL*‐*1* expression. Bisulphite sequencing of genomic DNA from monocytes identified allele‐specific demethylation that occurred at the T allele in contrast to the alternate C allele.^34^ SIRL‐1 is known to limit the production of reactive oxygen species in monocytes which play a role in inflammatory skin.^36^, ^37^ This led the authors to further investigate the significance of allele‐specific methylation in an AD patient cohort where they found a higher genotype frequency for the rs612529‐C allele in the AD patient group in comparison with healthy controls. The finding highlighted a CpG SNP and allele‐specific methylation bias as a potential risk factor for AD.

Differential methylation at CpG sites in AD has also been reported for the *KIF3A* gene encoding the cilia kinesin family member 3A structural protein.^38^, ^39^, ^40^ Two skin expression quantitative trait loci (eQTLs) were previously determined to be associated with differential *KIF3A* expression and AD susceptibility.^40^ Johansson et al sought to further determine the association of these SNPs with allergic rhinitis, asthma and AD in a paediatric disease biorepository of over 7000 children and a population‐based control group of 1020 children. rs11740584 (C) and rs2299007 (G) minor alleles are associated with lower *KIF3A* expressions in sun‐exposed and not sun‐exposed skins in GTEX.^41^ Stevens et al later determined that the minor alleles for rs11740584 and rs2299007 generated new CpG sites with 43% and 44% observed methylation, respectively.^42^ Individuals who were homozygous for the minor alleles exhibited higher methylation (78%), a striking comparison to homozygous reference allele individuals who were observed to have ≤10% methylation. Transepidermal water loss (TEWL) assays were next performed on human skin to determine whether methylated, and hence decreased *KIF3A*, resulted in skin barrier disruption. Higher TEWL measurements were found in individuals who genotyped for the minor alleles associated with increased methylation. Analysis of Kif3a‐deficient mice rigorously corroborated these findings for increased TEWL and an increased risk for developing AD. Together, the discovery in humans and mice revealed a significant role for the impact of DNA methylation for *KIF3A* on AD pathogenesis.

## 3 HISTONE MODIFICATIONS

The role of histone modifications has been explored in allergy (see review^6^), yet this knowledge is limited for AD. Specifically, increased enrichment for the transcriptionally active H3ac and H4ac histone marks was identified in allergic asthma patients and correlated with increased *IL13* in CD4+ T cells.^43^ A separate study discovered increased H3 histone acetylation at the promoter of *CXCL8* in airway smooth muscle cells and increased *CXCL8* expression in asthmatic patients and suggests histone acetyltransferase regulation in cells involved in allergic inflammatory pathways.^44^ Although additional studies are needed to further determine epigenetic mediated causation, the findings highlight the potential use of drugs that target histone modifications as new treatments for allergy.^6^

A more recent study sought to determine microbiome‐mediated epigenetic changes in an AD pathogenesis model.^45^ Butyric acid (BA) is a fermentation metabolite of the probiotic *Staphylococcus epidermidis* (*S*. *epidermidis*) in the skin, with inhibitory growth effects on *Staphylococcus aureus* (*S*. *aureus*), a known pathogenic bacteria in AD.^46^ BA is also a known inhibitor for histone deacetylase (HDAC) activity, resulting in increased histone acetylation and increased gene expression. Traisaeng et al demonstrated that mouse skin wounds, pretreated with BA and a BA derivative, BA–NH–NH–BA, and subsequent exposure to *S*. *aureus*, resulted in the suppression of *S*. *aureus* growth.^45^ Furthermore, human skin HaCaT keratinocytes treated with BA exhibited increased levels of transcriptionally active AcH3K9 (acetylation of histone H3 lysine 9) chromatin and decreased levels of the pro‐inflammatory interleukin, IL‐6. This exciting study highlighted a probiotic‐induced epigenetic response in host cells that was associated with reduced pathogenicity (growth) for *S*. *aureus*. It is clear that more studies are needed to further understand histone modifications in AD.

## 4 NON‐CODING RNA

A role for miRNAs (micro RNAs) in AD pathogenesis has also been an active area of investigation, as miRNAs post‐transcriptionally inhibit target mRNA transcripts via 3ʹ UTR binding and are also known to target HDAC and DNMT epigenetic regulators.^47^, ^48^, ^49^ miRNA microarray analysis identified decreased miR‐335 expression in AD lesions versus healthy control skin.^50^ miR‐335 transfection of N/TERT‐1 human keratinocyte cells resulted in increased transepithelial electric resistance and increased cornified envelope production in support of the protective effect of miR‐335 expression for healthy skin barrier maintenance. SOX6 was determined to be a direct target of miR‐335, as evidenced by decreased SOX6 transcript and protein expression in miR‐335‐transfected cells. The authors next found SOX6 interaction with SMARCA, a chromatin remodelling complex, based on coimmunoprecipitation and colocalization in keratinocytes, suggesting a role for the miR‐335/Sox6‐mediated gene regulation. This led the authors to examine the effects of HDAC activity on miR‐335 expression. HDAC inhibition in N/TERT‐1 cells by sodium butyrate (NaB) treatment resulted in significant miR‐335 upregulation. Together, these findings establish a premise for developing HDAC epigenetic regulators as new therapies to restore protective barrier effects imparted by miR‐335 expression in AD lesional skin.^50^

Differential miRNA expression was also reported in leukocytes from plasma obtained from an AD case‐control group.^51^ High‐throughput miRNA sequencing in 500 AD patients and 200 healthy controls identified 25 differentially expressed miRNAs with miR‐151a as the top miRNA that was upregulated in AD patients compared with healthy controls. *IL12RB2* was bioinformatically predicted to be a target of miR‐151a. Lentiviral transduction of miR‐151a in Jurkat cells resulted in decreased *IL12RB2* and other Th1 cytokines, IL‐2, IL‐12 and IFN‐γ expressions. Together, these findings identify miR‐151 as a key miRNA in AD pathogenesis.

Several other key miRNAs have also been reported in AD. miR‐124 is associated with inflammatory factors^52^, ^53^ and was reported to be downregulated in lesional AD skin compared with non‐lesional control patients.^54^ NF‐κB (p65), a key regulator of the inflammatory immune response, was found to be regulated by miRNA‐124. Overexpression of miR‐124 in normal human epidermal keratinocytes resulted in decreased p65 expression whereas inhibition of miR‐124 resulted in increased p65 expression. Additionally, a separate retrospective analysis of miRNAs obtained from umbilical cord serum revealed increased miR‐144 expression in children that developed AD symptoms at the age of 1 year, suggesting a miR‐144‐mediated epigenetic role in the pathogenesis of AD.^55^ This was further supported by miR‐144 transfected human keratinocytes resulting in increased hBD‐2 and SERPINB4, upstream activators of the pro‐inflammatory NF‐κB pathway. Bioinformatic analysis of microarray data from a GEO database of AD patients versus controls found 328 differentially expressed miRNAs with downregulated miRNAs associated with epidermal development and upregulated miRNAs associated with the immune response.^56^ Together, these studies reveal several miRNAs involved in AD inflammation via the NF‐κB pathway and a catalog of candidate miRNAs for which future functional studies are needed to further elucidate their role in AD pathogenesis.

Most recently, circular RNA (circRNA) expressions in the lesional and non‐lesional skins of patients with psoriasis or AD were also investigated in comparison with unaffected individuals.^57^ CircRNAs are non‐coding RNAs that exist as covalently bonded, closed loops as a result of non‐canonical splicing (see review^58^). These biomolecules have been proposed to act as microRNA “sponges,” thus indirectly regulating miRNA target genes,^59^, ^60^, ^61^, ^62^ and more recently for innate immune regulation.^63^ Motivated by this latter finding, high‐depth RNA‐seq analyses were performed on both psoriasis and AD lesional skin. circRNA expression profiles for each of these diseases were identified and were found to be distinguishable from healthy skin but with less circRNA abundance. Interestingly, ciRS‐7 was notably upregulated in AD lesional skin whereas circZRANB1 circRNA was increased in psoriasis and highlights the potential application of these circRNAs as discriminatory biomarkers for AD vs. psoriasis. As this study is the first to report circRNAs in AD, it paves the way for future investigations to further elucidate the impact of circRNAs on gene expression and cellular function in AD.

## 5 SUMMARY

In summary, studies to date have revealed key epigenetic differences in cell types that underlie AD pathogenesis. As of yet, AcH3K9 is the only histone modification analysis that has been reported for AD. As several studies have identified an association between histone modifications and allergy,^6^ this motivates future parallel studies for AD‐associated histone modifications. Investigations of eQTLs as CpG methylation switches for gene expression in AD have proven to be an exciting framework to further understand the impact of disease‐associated SNPs and motivates additional inquiries for other cataloged skin eQTLs that can be hypothesized as sites of differential DNA methylation. Additionally, the discovery for *NLRP2* hypermethylation and decreased expression in a birth cohort for persistent early‐onset AD and household smoke exposure highlights a unique opportunity to reverse disease‐associated methylation sites for AD prevention.^32^ Several studies have reported epigenetic differences in disease‐impacted keratinocytes. However, epigenetic changes have also been found in CD4+ CLA+ T cells and monocytes, yet more epigenetic studies are needed to comprehensively understand the relationships of other immune cell types and their epigenomes associated with AD. Moreover, differential expression of miRNAs that coincide with the DNA methylation and gene expression differences in these CD4+ CLA + T cells provides additional challenges to more fully elucidate the molecular underpinnings of AD. As we move forward, we must consider integrative and collaborative approaches to disentangle drivers versus passengers if we are ever to dissect the contribution of epigenetics to AD pathogenesis.

## CONFLICT OF INTEREST

The authors declare no conflict of interest.

## AUTHOR CONTRIBUTIONS

AS wrote the manuscript and prepare the graphical abstract. Both authors edited the manuscript.

## Data Availability

Data sharing is not applicable to this article as no data sets were generated or analysed during the current study.
